# Le DRESS syndrome secondaire aux antituberculeux: à propos d’un cas

**DOI:** 10.11604/pamj.2017.27.37.11663

**Published:** 2017-05-11

**Authors:** Siham Jridi, Rajae Azzeddine, Jamal Eddine Bourkadi

**Affiliations:** 1 Service de Pneumologie, Laboratoire PCIM, UCAM, Hôpital Arrazi, CHU Mohamed VI, Marrakech, Maroc; 2 Service de Pneumologie, Hôpital Moulay Youssef, CHU, Rabat, Maroc

**Keywords:** Syndrome d´hypersensibilité, antituberculeux, toxidermie, Hypersensitivity syndrome, antituberculosis drugs, toxidermia

## Abstract

Le syndrome d'hypersensibilité médicamenteuse ou Drug Rash with Eosinophilia and Systemic Symptoms ou DRESS syndrome est une toxidermie grave qui peut mettre en jeu le pronostic vital. Il faut y penser devant toute réaction cutanée après la prise des médicaments. Nous rapportons un cas clinique d'un patient âgé de 45 ans traité pour tuberculose pulmonaire TPM+ présentant un DRESS syndrome induit par les anti-bacillaires.

## Abstract (en)

Drug hypersensitivity syndrome or Drug Rash with Eosinophilia and Systemic Symptoms or DRESS syndrome is a severe and potentially life-threatening toxidermia. It should be suspected in patients developing cutaneous reaction following drug intake. We report the case of a 45-year old patient treated for pulmonary tuberculosis (TPM+) who developed DRESS syndrom induced by antibacillaries.

## Introduction

Le DRESS est une réaction médicamenteuse aiguë associant une éruption cutanée, une hyperthermie, des adénopathies diffuses, une éosinophilie sanguine et des atteintes viscérales variées. Il est potentiellement grave, avec une mortalité estimée à 10%. Nous rapportons un cas de DRESS syndrome aux antibacillaires, et nous insistons à travers cette observation sur la difficulté d'établir la relation cause effet lorsque plusieurs médicaments sont administrés de manière concomitante comme le cas du régime antituberculeux.

## Patient et observation

**Figure 1:**
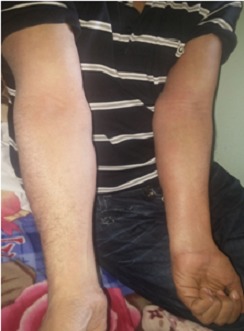
Lésions squameuses prurigineuses au niveau des bras et avant bras

**Figure 2:**
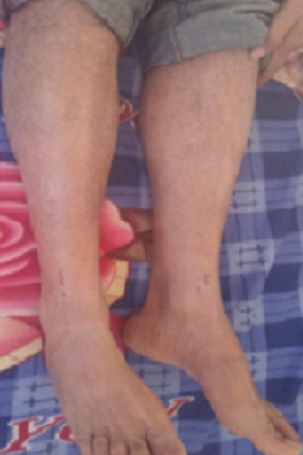
Lésions squameuses prurigineuses avec des lésions de grattage au niveau des jambs

**Figure 3:**
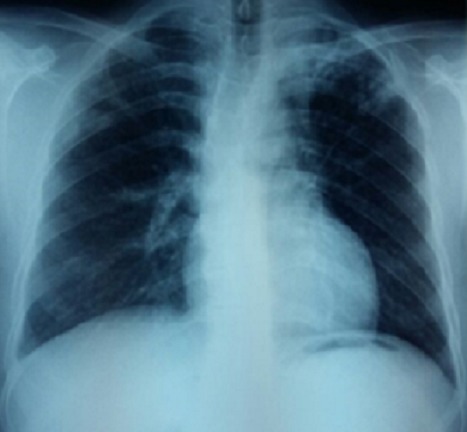
Radiographie thoracique du patient objectivant des infiltrats hilo-apicale gauche

C'est l'observation d'un patient âgé de 45 ans, tabagique chronique à 30 PA, sans autres ATCDS pathologiques particuliers, traité pour tuberculose pulmonaire confirmée à l'examen direct le 04/03/16 par la forme combinée de l'association rifampicine, Ethambutol, isoniazide et pyrazinamide (ERIP K4). Le patient a présenté depuis les 10 derniers jours de la phase d'attaque (à J50 du traitement) une fièvre, des œdèmes des deux membres inférieurs, avec apparition de lésions cutanées squameuses prurigineuses qui se sont généralisées à tous le corps (Figure 1, Figure 2). Ce qui a indiqué son hospitalisation. A l'admission dans notre service, le patient présente à l'examen clinique une fièvre à 38°, des œdèmes des membres inférieurs, des adénopathies jugulo-carotidiennes gauches, axillaires gauches et inguinales droites lenticulaires, l'examen pleuro-pulmonaire était sans particularité. Le traitement anti-bacillaire fut arrêté le 16/05/16. Le bilan biologique a objectivé une protéinurie positive à 1,02g/24h, une hyper-éosinophilie à 1430/UL, les sérologies HIV et hépatite virale B et C négatives. La recherche de BK dans les expectorations était négative. L'ionogramme sanguin et bilan hépatique étaient sans particularité La radiographie thoracique a montré des infiltrats hilo-apicale gauches (Figure 3), l'échographie abdomino-pelvienne n'a pas objectivé d'anomalie. Le diagnostic de DRESS a été retenu. La réintroduction du traitement anti-bacillaire a débuté le 08/06/16 après disparition des lésions cutanées, par la Rifampicine faite sur 3 jours (150mg, 300mg, 600mg) sans incidents. Après la réintroduction progressive de l'Isoniazide sur 3 jours (75mg, 150mg, 300mg), le patient a présenté le 2^ème^ Jour à 150mg de l'isoniazide des œdèmes des membres inférieurs avec prurit généralisé, une substitution de l'isoniazide par l'Ethambutol a été faite avec apparition d'un prurit généralisé important dès son introduction, la conduite à tenir a consisté en l'arrêt du traitement (Rifampicine+ Ethambutol) jusqu´à disparition des lésions avec mise du patient sous traitement symptomatique. Le patient a été remis le 10/06/16 sous Rifampicine à dose complète 600 mg + Lévofloxacine 750 mg, l'évolution a été marqué le 04/07/16 par la réapparition du prurit et des œdèmes des membres inférieurs d'où l'arrêt du traitement et la mise sous Isoniazide + Lévofloxacine le 10/07/16. Le 20/07/16 et après une bonne tolérance du traitement prescrit, le patient est réadmis pour réintroduction de l'Ethambutol, prévu sur 3 jours (400mg, 800mg, 1200mg), à 800 mg le patient a présenté des œdèmes des membres inférieurs avec un prurit généralisé, l'Ethambutol fut arrêté et le patient a été remis après disparition des lésions sous isoniazide et lévofloxacine sans incident avec prolongation de la durée du traitement de 6 mois. La responsabilité de Rifampicine et de l'Ethambutol est fortement suspectée.

## Discussion

Le DRESS syndrome est un syndrome d'hypersensibilité médicamenteuse [1]. Le terme « DRESS syndrome » a été utilisé pour la première fois en 1996 par Bocquet et al. [1]. Les symptômes surviennent après 2 à 6 semaines du début du traitement, dominés le plus souvent par les lésions cutanées et hépatiques. Sur 172 cas de DRESS syndrome, 44 médicaments sont répertoriés avec un seul cas lié à la streptomycine [2]. En 2012, un cas du DRESS syndrome à l'éthambutol et à la rifampicine a été notifié [3] comme c'est le cas de notre observation, un autre cas de DRESS syndrome aux anti-bacillaires notamment à l'ethambutol et l'isoniazide avec un doute sur la rifampicine a été rapporté par Bouyad et al [4] et un autre cas a été noté par Iraqi et al [5] à l'éthambutol, à l'isoniazide et à la pyrazinamide. La majorité des patients présentant le DRESS syndrome ne sont initialement pas correctement diagnostiqués [6]. Il existe néanmoins plusieurs consensus sur les critères diagnostiques de DRESS syndrome proposés par certains auteurs [7, 8]. En tenant compte de critères de Kardaun (Tableau 1), notre patient présente 4 critères positifs; l'hyper-éosinophilie, fièvre à 38.5°, des adénopathies dans 3 territoires et le rash aigu. Selon les critères du groupe japonais (Tableau 2), notre patient présente 5 critères, l'exanthème maculopapuleux > 3 semaines, les manifestations cliniques persistantes plus de 2 semaines, les poly-adénopathies, l'hyper-éosinophilie et la fièvre. La prise en charge du DRESS, en dehors de l'arrêt du médicament incriminé, n'est pas bien codifiée. Elle dépendra des éléments clinico-biologiques et évolutifs. Ainsi dans certains cas, aucun traitement n'est nécessaire, alors que dans d'autres cas un traitement par corticothérapie générale et/ou immunoglobulines intraveineuses et/ou les antiviraux (ganciclovir) peut être nécessaire [9]. S'il est absolument nécessaire d'utiliser un médicament potentiellement responsable, il devrait être administré avec prudence en commençant par une petite dose [3]. La survenue de poussées évolutives à distance n'est pas rare même en absence de toute nouvelle prise médicamenteuse justifiant une surveillance biologique prolongée [9].

**Tableau 1:** Critères RegiSCAR d’inclusion de DRESS syndrome

Hospitalisation	
Suspicion de lien entre la réaction et un médicament	Adénopathie dans au moins 2 sites distincts*
Rash aigu*	Anomalies de la formule sanguine (lymphopénie ou lymphocytose*, éosinophilie*, thrombocytopénie*)
Fièvre > 38 _C*	Trois critères sur les quatre marqués d’une étoile sont nécessaires pour poser le diagnostic

**Tableau 2:** Critères du groupe japonais de consensus du Drug-induced Hypersensitivity Syndrome (DiHS) ou DRESS syndrome

Rash maculopapuleux apparaissant plus de 3 semaines après le début du traitement
médicamenteux incriminé
Persistance des symptômes 2 semaines après l’arrêt du traitement médicamenteux incriminé
Fièvre > 38 _C
Troubles de la fonction hépatique (ALAT > 100 U/L)
Anomalies leucocytaires :
Hyperleucocytose (> 11×109/L)
Lymphocytes atypiques (> 5 %)
Hyperéosinophilie (> 1,5× 109/L)
Lymphadénopathie
Réactivation HHV-6
Sept critères présents = DiHS typique. Cinq premiers critères présents = DiHS atypique

## Conclusion

le DRESS syndrome du aux anti-bacillaires est rare, il ne doit pas être méconnu. La conduite à tenir n'est pas codifiée. Il faut rapporter plus de cas dans le but d'instaurer et codifier une meilleure prise en charge de ces patients.

## Conflits d’intérêts

Les auteurs ne déclarent aucun conflits d'intérêts.
